# High-dose atorvastatin reduces the risk of cardiovascular events in patients with percutaneous coronary intervention

**DOI:** 10.18632/oncotarget.19701

**Published:** 2017-07-31

**Authors:** Changqing Lu, Helei Jia, Zhentao Wang

**Affiliations:** ^1^ Department of Emergency, Henan Province Hospital of Traditional Chinese Medicine, Zhengzhou, Henan Province 450002, China; ^2^ Department of Cardiovascular Disease, Henan Province Hospital of Traditional Chinese Medicine, Zhengzhou, Henan Province 450002, China

**Keywords:** percutaneous coronary intervention, atorvastatin, intervention, meta-analysis, randomized controlled trials

## Abstract

We systematically searched in PubMed, Web of Science, Embase and China National Knowledge Infrastructure from the inception to March 31, 2017, identified relevant trials about efficacy of high-does Atorvastatin for patients with percutaneous coronary intervention. Twelve studies with the number of 2801 patients were included in the meta-analysis. Compared with control group, high-does Atorvastatin significantly reduced the risk of myocardial infarction in patients with percutaneous coronary intervention (Relative risk =0.62, 95% confidence interval: 0.49-0.78), with low level of heterogeneity (I^2^=22.6%, P=0.228). Nine studies with 2248 patients reported the adverse cardiovascular events. A fixed-effect model was applied. Compared with control group, patients with high-does Atorvastatin taken, the risk of adverse cardiovascular events was degraded by 65% (Relative risk, RR=0.65, 95% confidence interval (CI): 0.50-0.84), which was confirmed by trial sequential analysis as the cumulative Z curve entered the futility area. The subgroup analyses found that decreased risks of myocardial infarction among trails (RR=0.64, 95%CI: 0.50-0.83, RR=0.55, 95%CI: 0.34-0.88). Egger and Begg’s test found no publication bias (t=-1.670, *P*=0.129; Z=1.560, *P*=0.119). The use of high-dose Atorvastatin could reduce the risk of myocardial infraction and cardiovascular adverse events in patients with percutaneous coronary intervention. High-dose Atorvastatin was recommended as an adjunct to aid percutaneous coronary intervention.

## INTRODUCTION

Percutaneous coronary intervention (PCI) is common invasive procedure in clinical setting, especially for patients with coronary artery disease. Although many studies have suggested that this procedure was safe and was related to decreased risk of some complications, and myocardial infarction, as evaluated by cardias function index, there was a still chance to occur in 5%-40% of patients according to different definition [1, 2]. These complications have great negative effect on clinical prognosis after intervention treatment. To overcome this issue, researchers had focused on various clinical strategies to reduce the risk of cardiovascular adverse outcomes during PCI such as beta-blockers, antiplatelet agent and so on [3, 4].

Previous experimental studies indicated that statins had cardioprotective effects in the animal model of ischemia-reperfusion [5]. Some clinical results also demonstrated that taking statins before PCI treatment could significantly decrease the incidences of some complications during intervention and cardiovascular adverse events in patients who underwent PCI [6]. However, these studies focused on observational design with limited evidence level. Results from randomized controlled trials remained inconsistent. Single randomized trial still had some limitations such as sample size, study population, drug types, low statistical power, which were not enough to assess clinical outcomes [7–9]. The recent meta-analyses on this topic included several different types of statins [10]. We were not sure that one or more statins exerted effects on clinical outcomes. Moreover, four recent trials with adequate statistical power have been published [9, 11–13]. Evidences about this topic were required to be updated. Therefore, we conducted a latest meta-analysis to evaluate efficacy of high-dose Atorvastatin on clinical outcomes in patients with percutaneous coronary intervention, and we further used trial sequential analysis to confirm whether the present results were robust.

## RESULTS

### Trial selection

**Figure 1:**
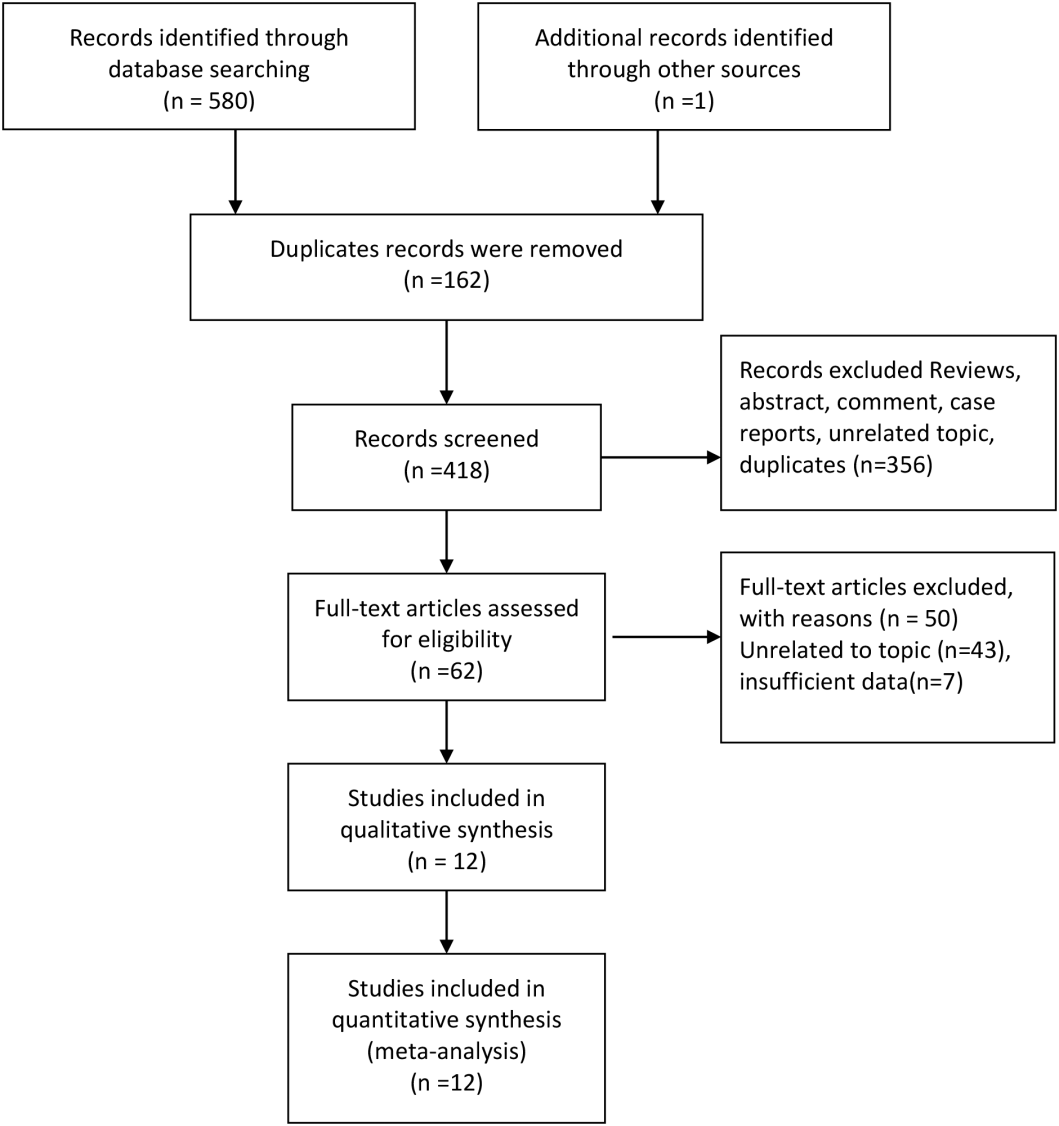
Selection of trials for meta-analysis

The Figure 1 presented the process of trials selection. We identified 580 records through database searching, and identified one record through retrieving the references lists of articles and review. 418 articles were left for titles and abstracts screened after 162 duplicates records were removed. We excluded comments, case reports, unrelated topics, and 62 articles were planned for full-text scanning. Fifty studies were excluded for insufficient data and unrelated topics. Finally, twelve studies were included in qualitative and quantitative synthesis [7–9, 11–19].

### General characteristics of trails

The general characteristics of results were summarized in Table 1. These studies were published from 2004 to 2014. Sample size ranged from 42 to 668, with the number of 2801 patients. The duration of follow-up ranged from 1 to 45 months. Of these included studies, six studies reported patients with stable angina pectoris solely, four were conducted in patients with non-ST segment elevation acute coronary syndrome. One included unstable and stable angina pectoris, and one included stable angina pectoris and not-ST segment elevation acute coronary. The intervention in trials was taking Atorvastatin preoperative, and control group received placebo (routine treatment). Four studies reported history of Atorvastatin in study population and eight studies reported no history of Atorvastatin. The does and time settings were different among trials. All studies were performed among adult.

**Table 1:** General characteristic of included randomized controlled trials in the meta-analysis

Author	Year of publication	Type of diseases	History of drug	intervention		Follow-up time	Sample size		Outcomes
				Trial	Control		Trial	Control	
Pasceri	2004	stable angina pectoris	No	80mg/day for a week, preoperative	Placebo	30 days	76	77	1,2
Patti	2007	non-ST segment elevation acute coronary syndrome	No	80mg, 12h+40mg, 2h preoperative	Placebo	30 days	86	85	1,2
Kinoshita	2007	stable angina pectoris	No	5-20mg/day, 2 weeks, preoperative	Placebo	6 months	21	21	1,2
Briguori	2009	stable and unstable angina pectoris	No	80mg, 24h preoperative	Placebo	-	338	330	1,2
Di	2009	stable angina pectoris, non-ST segment elevation acute coronary syndrome	Yes	80mg/day, 12h+40mg, 2h preoperative	Placebo	30 days	192	191	1,2
Toso	2011	stable angina pectoris	No	80mg, 48h preoperative	Placebo	-	77	84	1,2
Veselka	2011	stable angina pectoris,	No	80mg, 2 days preoperative	Placebo	45 months	100	100	1
Yu	2011	non-ST segment elevation acute coronary syndrome	No	80mg, 12h+40mg, 2h preoperative	Placebo	1 month	41	40	1,2
Zemanek	2013	stable angina pectoris	Yes	80mg/day for a week, preoperative	Placebo	-	100	102	1.2
Li	2013	non-ST segment elevation acute coronary syndrome	Yes	preoperative	Placebo	1 month	106	109	1
Jang	2014	non-ST segment elevation acute coronary syndrome	No	80mg, 12h+40mg, 2h preoperative	Placebo	1 month	163	172	1,2
Nafasi	2014	stable angina pectoris,	Yes	80mg, 24h preoperative	Placebo	-	95	95	1

### Assessment of quality

**Figure 2:**
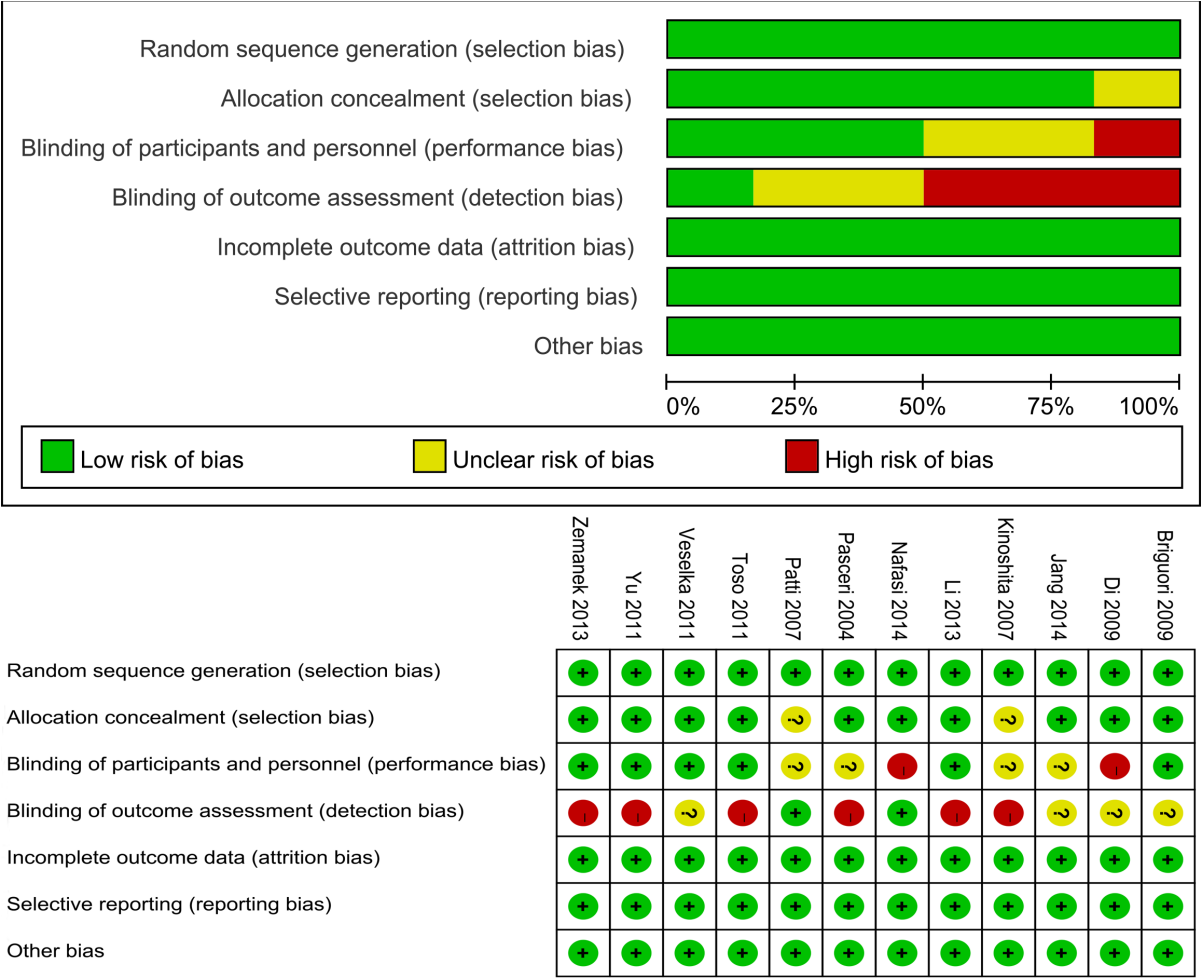
Proportion and summary of bias risk

The Figure 2 presented judgements about each risk of bias item for each included study and each risk of bias item presented as percentages across all included studies. Seven studies were considered as being unclear risk, and eight studies as being high risk because of blinding application. Randomized sequence generation were adequate among these studies. However, blinding application was extremely difficult and usually infeasible for these trials, we supposed that the outcomes was less influenced by the lack of blinding. Thus, the quality of included trials were quite high.

### Pooled results

**Figure 3:**
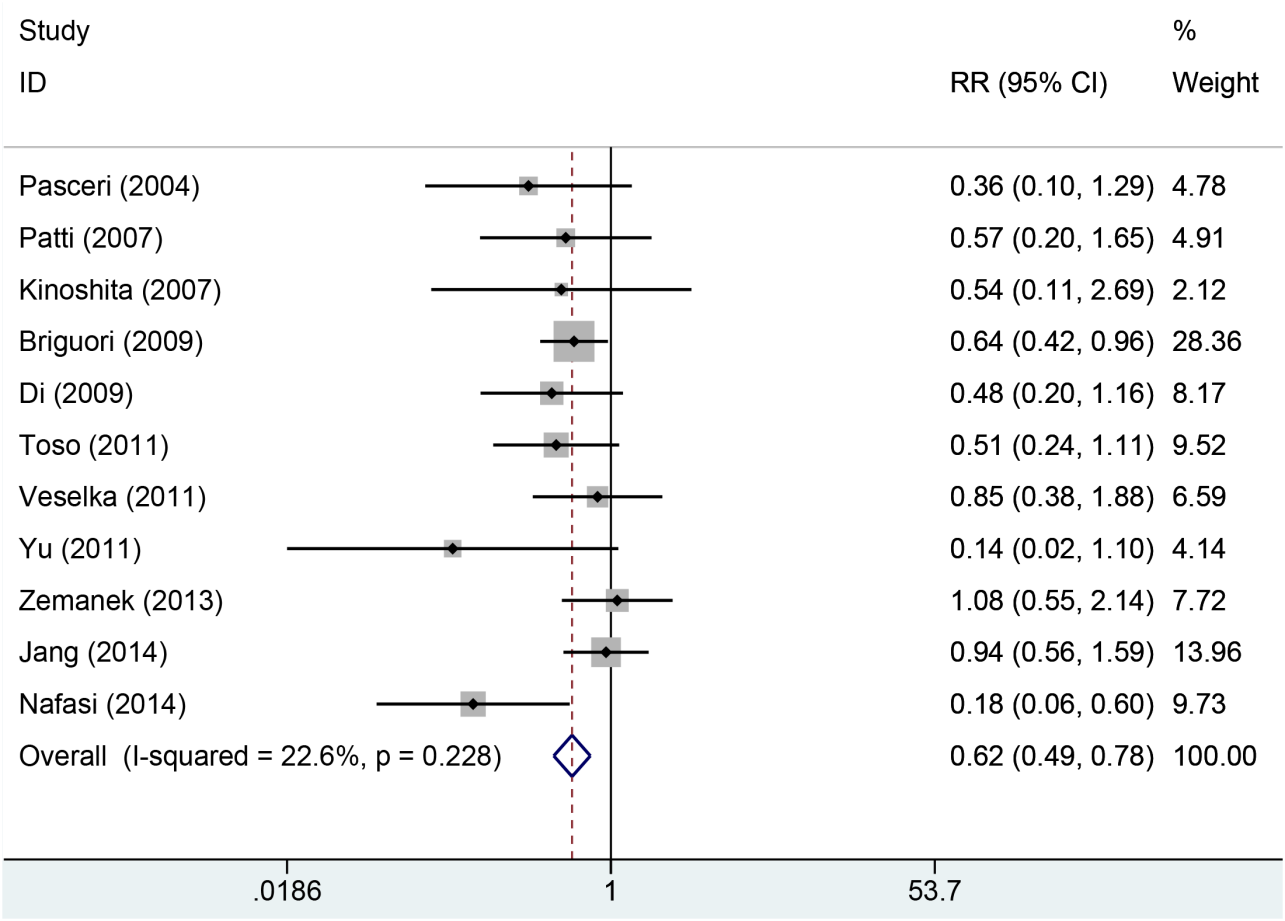
Forest plot of incidence of myocardial infraction of PCI in two groups

**Figure 4:**
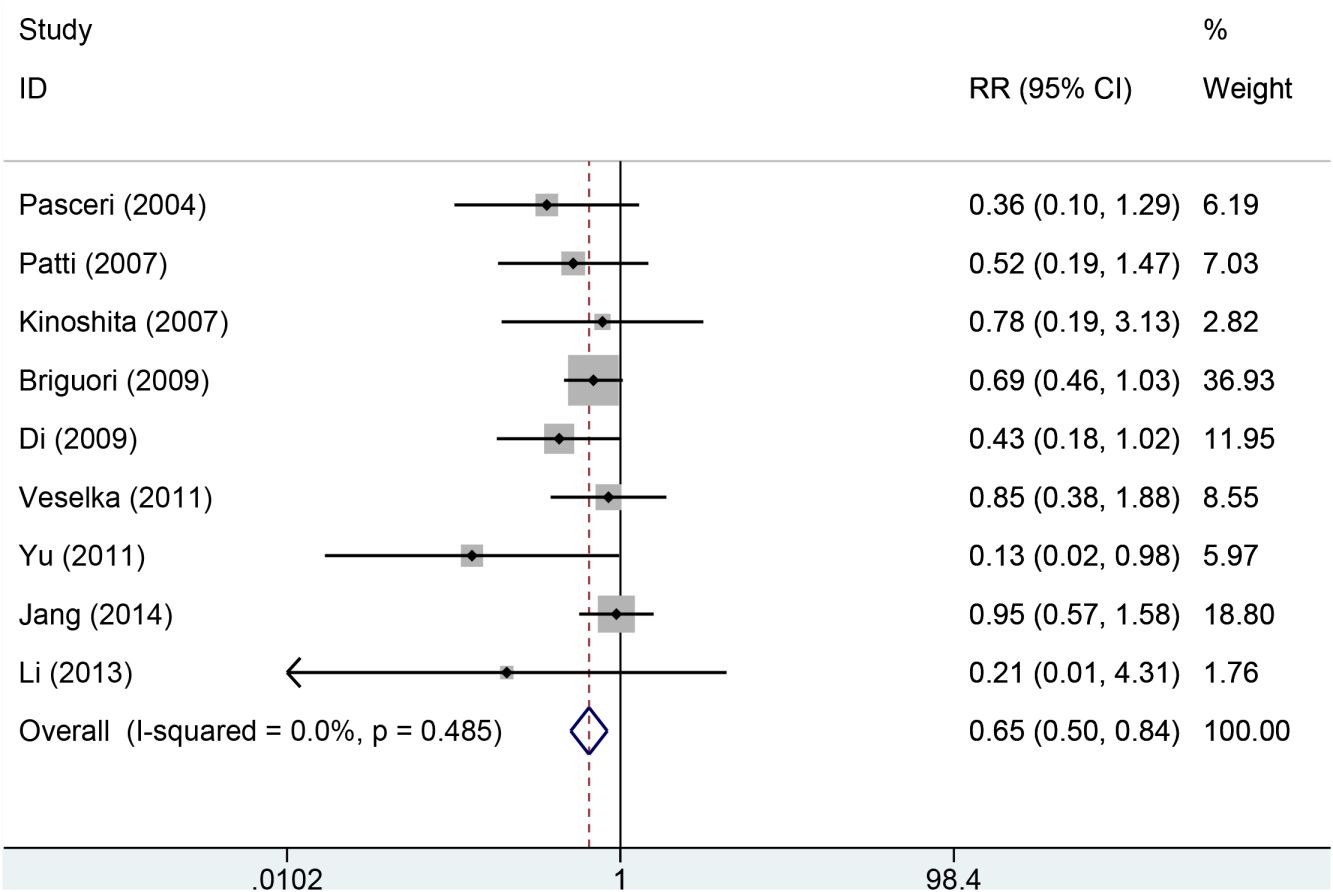
Forest plot of comparison on adverse outcomes between two groups

Eleven studies with 2850 patients reported the incidences of myocardial infarction. Compared with control group, high-does Atorvastatin significantly reduced the risk of myocardial infarction in patients with percutaneous coronary intervention (Relative risk (RR) =0.62, 95% confidence interval (CI): 0.49-0.78, Figure 3), with low level of heterogeneity (I^2^=22.6%, *P*=0.228). Trial sequential analysis of 12 trials (black square filled icons) illustrating that the cumulative z curve crossed both the conventional boundary for benefit and the trial sequential monitoring boundary for benefit, establishing sufficient and conclusive evidence and suggesting that further trials are not required. A diversity-adjusted required information size of 3,235 patients was calculated using a 0.05 (two-sided) and b =0.20 (power of 80%), an anticipated relative risk reduction of 20%, and an event proportion of 45% in the control arm. Nine studies with 2248 patients reported the adverse cardiovascular events. No significant heterogeneity was found for this estimation (I^2^=0.0%, *P*=0.458), and a fixed-effect model was applied. Compared with control group, patients with high-does Atorvastatin taken, the risk of adverse cardiovascular events was degraded by 65% (RR=0.65, 95%CI: 0.50-0.84, Figure 4).

**Figure 5:**
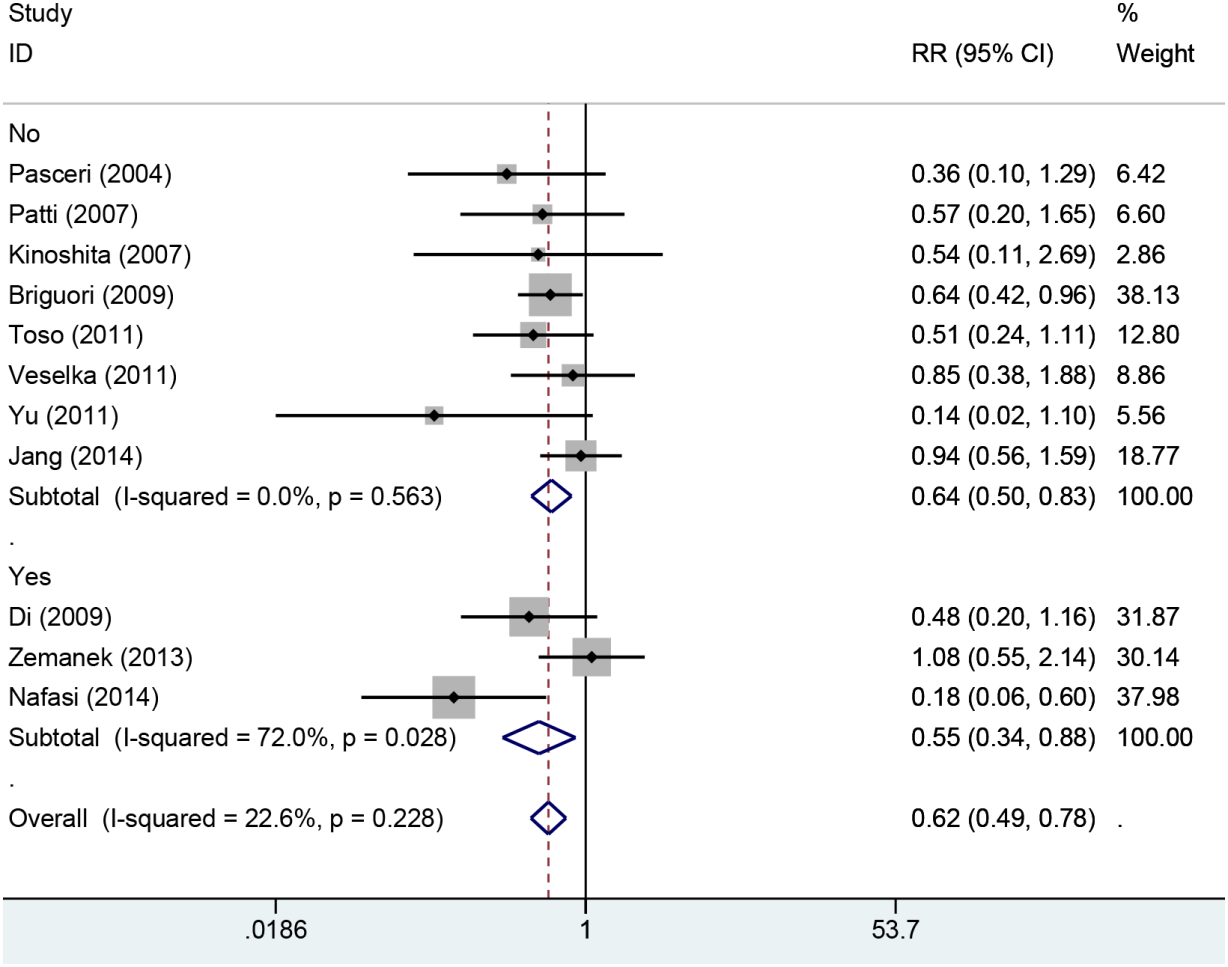
Forest plot of myocardial infraction for patients with/without taking Atorvastatin

We also conducted subgroup according to history of taking Atorvastatin. Four study reported the history of taking Atorvastatin, and eight studies did not report data. The results from fixed-effect model found decreased risks of myocardial infarction among trails (RR=0.64, 95%CI: 0.50-0.83, RR=0.55, 95%CI: 0.34-0.88 Figure 5). TSA indicated that the cumulative Z curve crossed the futility boundary and entered the futility area, building sufficient and conclusive evidence and showing that further trials were not required (Figure 7).

**Figure 6:**
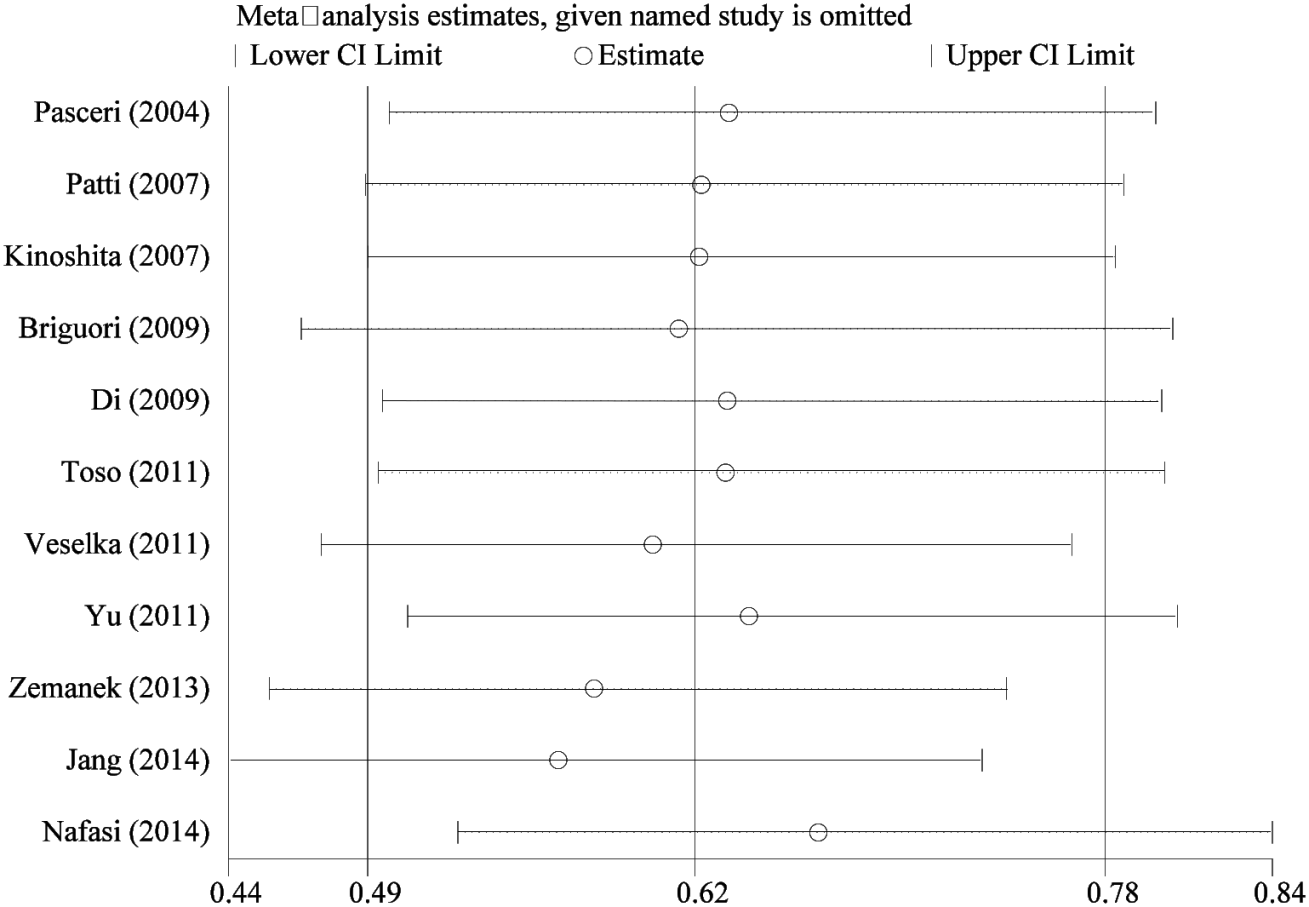
Sensitivity analysis of pooled results of myocardial infraction incidence

**Figure 7:**
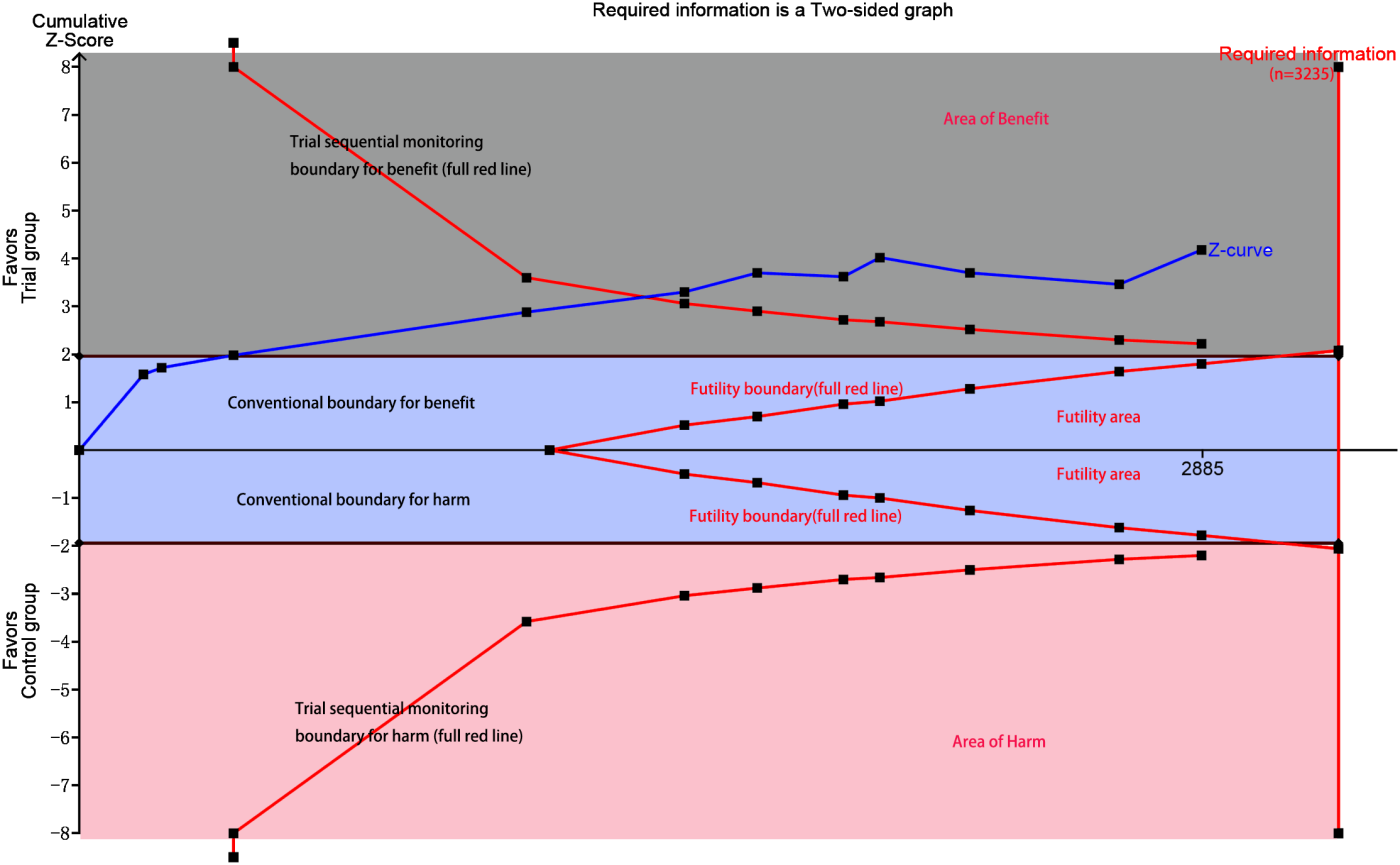
Trial sequential analysis of included trials comparing trial group and with control group for incidence of myocardial infraction (X axis = number of patients randomized; Y axis=cumulative z score; horizontal green dashed lines=conventional boundaries (upper for benefit, z score=1.96; lower for harm, z score=–1.96; two-sided P=0.05); sloping red lines with black filled circles=trial sequential monitoring boundaries calculated accordingly; blue line with black filled squares=z curve; vertical red line=required information size calculated accordingly; upper gray rectangle: area of benefit; middle blue rectangle=futility area; lower red rectangle=area of harm)

### Sensitivity analyses and publication bias

To explore the stability of pooled results, we conducted sensitivity analyses through excluding single study each time. The Figure 6 presented results of sensitivity analyses, the estimations with one study excluded still fallen into the 95%CI range of overall pooled result. This point indicated the combined results was reliable and stable. We assessed the publication bias by inspecting funnel plot and qualitative statistical test. The Egger and Begg test found no publication bias (t=-1.670, *P*=0.129; Z=1.560, *P*=0.119). The funnel plot given slight asymmetry (Supplementary Material 1).

## DISCUSSION

We conducted systematical search and comprehensive analyses, and our meta-analysis suggested that high-does Atorvastatin could reduce incidence of perioperative myocardial infarction and decrease the risk of cardiovascular adverse events. The evidence of benefit was confirmed in subgroup analysis, and trial sequential analysis showed that no further trials were required and the results were conclusive. Although some findings of our meta-analysis were the same as the previous study report [10], differences between our results and previous studies need to be addressed. The previous meta-analysis focused on different kinds of statins, and our study only care about one type of drug. As we all known, the interaction within different agents could exist [20]. It was not certain which one or several of them exerted effect. The present study excluded this confounding factor, and make more reliable conclusion. That was the most distinguishing characteristic of the two studies. Meanwhile, our study included latest publication about Atorvastatin usage with more robust statistical power. According to our trial sequential analysis, at least 3235 patients were required, the present study met this required information. The sufficient and conclusive evidence may help medical staffs make better clinical decisions.

Potential benefits associated with PCI was not fully understood. Previous study reported that short-term pretreatment with atorvastatin could improve outcomes in patients with acute coronary syndromes [15]. Later study also reported the same results [17]. Recent results found high-dose atorvastatin reduced the risk of myocardial infarction but without benefit regarding contrast-induced nephropathy [18]. The following reported found that short-term pretreatment with atorvastatin significantly only reduced procedural myocardial injury in early PCI [19]. The present study gave conclusive results through comprehensive and systematical analyses. Myocardial infarction perioperative period was one of common complications during PCI [9]. The myocardial infarction could happen when lateral branches occlusion, embolism of distal, coronary dissection appeared. Although cardiac function did not change a lot, the mortality was high. It was suggested that the elevation of creatinine kinase isoenzyme was associated with increased risk of mortality, and the stains could the incidences of myocardial infarction perioperative [13]. The atorvastatin belonged to one of inhibitors of 3-hydroxy-3-methylglutaryl coenzyme A, this type of agent could improve the endothelial dysfunction and increase stability of atherosclerotic plaque because of its characteristics of anti-inflammatory, anti-thrombotic and antioxidant. Atorvastatin was an important agent for preventing myocardial infarction perioperative. Ray found that high-does’ atorvastatin could reduce the incident of cardiovascular adverse outcomes compared with control group, the risk was degraded by 28% after 30 days and 6% after 2 years [21]. We had planned to conduct subgroup analysis according the type of angina pectoris. However, significant difference was found for the whole but not subgroup. We supposed that the few sample sizes were the reason. The different result was found when we conducted subgroup analysis according to history of atorvastatin.

The main strength of our meta-analysis was in accordance with the Preferred Reporting Items for Systematic Reviews and Meta-Analyses statement guidelines. Applying trials sequential analysis assessed the effect of randomized errors and sufficiency of sample size. Several study limitations should be addressed. Firstly, the given does and time point of atorvastatin were different. Some interventions were conducted two hours before operation, and some 24 hours before operation. This difference could make some biased estimations. Secondly, the follow-up duration of some studies was not long enough, it was possible that the expected outcomes did come up because of the short follow-up periods. This situation may not happen because our sensitivity analyses indicated that results were still stable when excluding these studies. Thirdly, some information of some study was not incomplete such as sex. We cannot conduct some such a subgroup. Finally, almost of all included studies did not apply blinding in the study protocol, and some detection bias may exist.

In conclusion, our results found that high-dose atorvastatin can effectively reduce the risk of perioperative myocardial infarction and cardiovascular adverse outcomes. The trial sequential analysis confirmed the effect. High-dose atorvastatin was recommended as adjunct before percutaneous coronary intervention.

## MATERIALS AND METHODS

We perform this meta-analysis by following the Preferred Reporting Items for Systematic Reviews and Meta-Analyses statement guidelines (Supplementary Material 2) [22]. No ethical approve was required for the current meta-analysis based on published studies.

### Literature search

We conducted a systematical online search in PubMed, Web of Science, Embase and China National Knowledge Infrastructure from the inception to March 31, 2017. We conducted electronic searches using exploded medical subject headings terms and corresponding Keywords: Atorvastatin, percutaneous coronary intervention, PCI, stents, angioplasty, randomly, randomized controlled trials, and RCT. We restricted the language in Chinese and English. To obtain the potentially eligible trials, we also retrieved the reference lists of articles and reviews. The latest publication was included for several reports of same study.

### Selection criteria

Two investigators independently performed literature search, excluded duplicate publication, scanning titles and abstracts, and identified studies as included or not. We downloaded full-text of potential studies and confirmed whether studies could be included in the analyses. The included studies must meet the following criteria: (1) Study design: randomized controlled trials from Chinese and English; (3) Study population: Patients who received PCI, including stable angina pectoris, unstable angina, non-ST segment elevation acute coronary syndrome, myocardial infraction, regardless of age and mode of administration; (4) Intervention: trials group received Atorvastatin and routine treatment, and control group received routine treatment (aspirin, clopidogrel, heparin and so on). (5) Outcomes: morbidity of myocardial infraction, and incidences of adverse cardiovascular events.

### Data extraction

We used a standard excel sheet to extract relevant information. One of the investigators performed data extraction and checked by other authors. The following information was extracted: the first author, year of publication, types of diseases, sample size, intervention, duration of follow-up, and outcomes.

### Assessment of quality

We assessed the quality of included studies via the risk of bias tool recommended by Cochrane handbook [23]. This assessment tool consisted of the following seven items: random sequence generation; allocation concealment, blinding including study design and outcome assessment, selected reported data, incomplete data, and other potential bias. We identified each item as low risk, high risk or unclear risk according to reported results of each study.

### Statistical analysis

For current study, the relative risk with 95% confidence intervals were calculated for dichotomous. We used the Chi-square test and I^2^ statistic to assess the heterogeneity across studies. When heterogeneity was found with P > 0.10 or I^2^> 50%, a random-effect model was used, or a fixed-effect model was used [24]. We pooled outcome data using a random-effect model because of potential clinical heterogeneity. Sensitivity analyses was conducted by excluding individual study each time. To explore the possible influences of various factors, we also set some subgroup analyses. Publication bias was evaluated by visually inspecting a funnel plot, and assessed using Begg’s and Egger test [25, 26]. All statistical analyses were completed on Stata 12.0 and RevMan 5.3 software platform. P<0.05 was considered as statistical significance, except specified setting.

## SUPPLEMENTARY MATERIALS





## Abbreviation

PCI: percutaneous coronary intervention
