# Supplementary material for: High-dose atorvastatin reduces the risk of cardiovascular events in patients with percutaneous coronary intervention
Source: Oncotarget. 2017 Jul 31;8(41):70356–65. doi: 10.18632/oncotarget.19701 (PMC5642560; doi:10.18632/oncotarget.19701)
Supplement: Supplementary file 1 [file oncotarget-08-70356-s001.pdf]

High-dose atorvastatin reduces the risk of cardiovascular events in patients with percutaneous coronary intervention

SUPPLEMENTARY MATERIALS

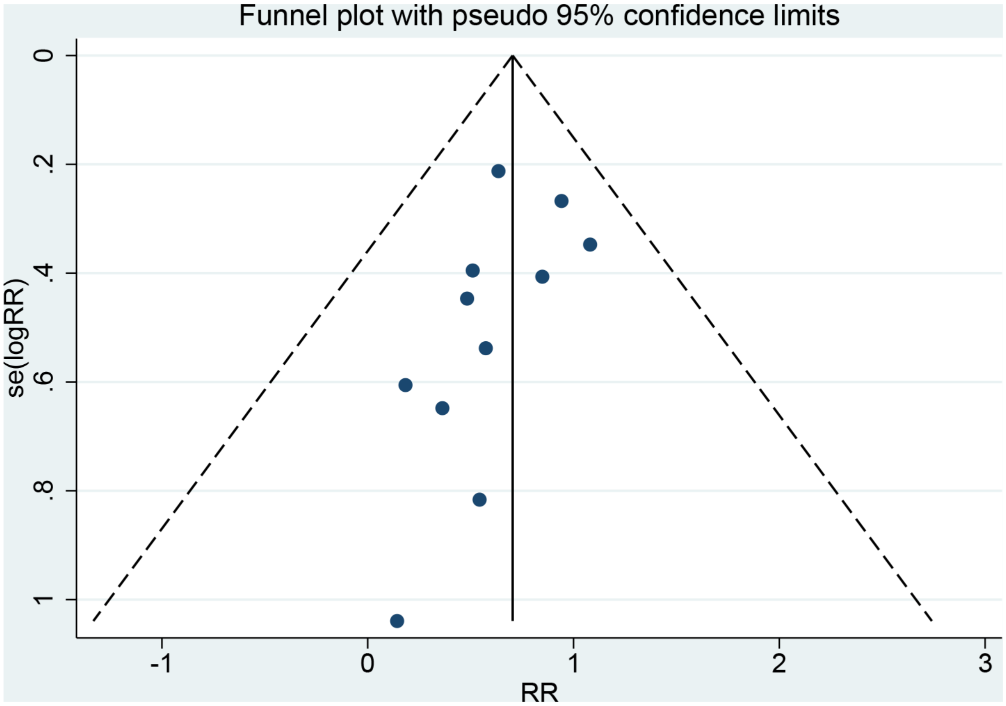

Supplementary Material 1: Funnel plot to evaluate the publication bias.

Supplementary Material 2: PRISMA checklist

See Supplementary File 1
